# Photosymbiosis for Biomedical Applications

**DOI:** 10.3389/fbioe.2020.577204

**Published:** 2020-10-06

**Authors:** Myra N. Chávez, Nicholas Moellhoff, Thilo L. Schenck, José Tomás Egaña, Jörg Nickelsen

**Affiliations:** ^1^Molecular Plant Science, Department Biology I, Ludwig-Maximilians-Universität München, Munich, Germany; ^2^Division of Hand, Plastic and Aesthetic Surgery, University Hospital, Ludwig Maximilian Universität München, Munich, Germany; ^3^Institute for Biological and Medical Engineering, Schools of Engineering, Biological Sciences and Medicine, Pontificia Universidad Católica de Chile, Santiago, Chile

**Keywords:** transgenic microalgae and cyanobacteria, photosynthetic oxygen, hypoxia, regenerative medicine, tissue engineering, recombinant proteins and monoclonal antibodies

## Abstract

Without the sustained provision of adequate levels of oxygen by the cardiovascular system, the tissues of higher animals are incapable of maintaining normal metabolic activity, and hence cannot survive. The consequence of this evolutionarily suboptimal design is that humans are dependent on cardiovascular perfusion, and therefore highly susceptible to alterations in its normal function. However, hope may be at hand. “Photosynthetic strategies,” based on the recognition that photosynthesis is the source of all oxygen, offer a revolutionary and promising solution to pathologies related to tissue hypoxia. These approaches, which have been under development over the past 20 years, seek to harness photosynthetic microorganisms as a local and controllable source of oxygen to circumvent the need for blood perfusion to sustain tissue survival. To date, their applications extend from the *in vitro* creation of artificial human tissues to the photosynthetic maintenance of oxygen-deprived organs both *in vivo* and *ex vivo*, while their potential use in other medical approaches has just begun to be explored. This review provides an overview of the state of the art of photosynthetic technologies and its innovative applications, as well as an expert assessment of the major challenges and how they can be addressed.

## Introduction

Molecular oxygen is essential for cell metabolism and energy production. Consequently, the adaptive response to oxygen fluctuations is one of the most important coupling mechanisms in animal cells and tissues, as revealed by the ground-breaking discoveries recognized by the 2019 Nobel Prize in Physiology and Medicine. In intact tissues, oxygenation is provided by the circulatory system. Loss or absence of blood perfusion eventually leads to severe tissue necrosis—and even localized damage to the microvasculature through surgical interventions, trauma, diabetes, compromised vasculature integrity or peripheral arterial disease—can lead to tissue hypoxia and subsequent functional impairment (Gordillo et al., 2003; Sen, 2009).

Moreover, oxygen is of central importance for tissue regeneration and a crucial mediator of wound healing, since it is involved in every stage of the reparative process, including collagen synthesis, angiogenesis, and epithelialization (Rodriguez et al., 2008; Sen, 2009; Thom, 2011; Eisenbud, 2012). Besides, adequate supplies of oxygen to wound sites are required not only to maintain high ATP levels in hypermetabolic proliferating cells, but also for the generation of reactive oxygen species, which act as key signaling molecules in regenerative pathways and play a central role in preventing wound infections. The implications of hypoxia for tissue subsistence and repair have been reviewed extensively, and recent studies have focused on boosting oxygen levels in wounds by providing alternative sources of oxygen, such as topical oxygen therapy, oxygen-releasing wound dressings, supplemental oxygen therapy or hyperbaric oxygen chambers. However, these measures have shown limited success and have not become part of routine clinical practice (Kim et al., 2007; Kimmel et al., 2016; De Smet et al., 2017; Sanford et al., 2018).

On the other hand, certain clades of organisms have acquired oxygen-producing capacities through the incorporation of photosynthetic microalgae or cyanobacteria. Stimulated by these examples of photosymbiotic relationships in nature, the emergence of photosynthetic biotechnology represents a major opportunity to address hypoxia-related issues in regenerative medicine, tissue engineering and even cancer treatment. Furthermore, the combination of biotechnological approaches with photosynthetic organisms opens up the prospect of reversing the life-threatening impact of a hypoxic microenvironment in other medical conditions such as stroke, myocardial infarction and organ transplantation (Table 1). In the following, we describe the principles and assumptions behind the photosynthetic technologies devised so far, summarize the main findings and review their shortcomings, with the aim of promoting their further development and extending the scope of their applications.

**TABLE 1:** Organisms employed for photosynthetic oxygenation in medical applications.

**Species**	**Classification**	**Relevant attributes**	**Biomedical application**	**Illumination**	**References**
*C. reinhardtii*	Unicellular eukaryotic green alga of the genus *Chlamydomonas*	Model organism in areas such as flagellar function, photobiology, photosynthesis and recombinant protein synthesis. Optimal cell growth between 20 and 25°C in defined salt-based liquid or agar media at neutral pH	• Proof-of-principle of alga-vertebrate symbiosis Photosynthetic dermal scaffolds. • Photosynthetic sutures. • Photosynthetic gene therapy for expression and delivery of recombinant proteins	Constant illumination, white light (1,500–2,500 lux, 72.5 μmol m^–2^ s^–1^)	Hopfner et al., 2014; Alvarez et al., 2015; Schenck et al., 2015; Chávez et al., 2016; Centeno-Cerdas et al., 2018
			• “Green bioprinting” of 3D alginate photosynthetic scaffolds	Constant illumination or 14/10 h L/D cycles, warm−white light−emitting diode panel (20 μmol m^–2^ s^–1^)	Krujatz et al., 2015; Lode et al., 2015
*C. littorale*	Unicellular eukaryotic green alga of the genus *Chlorococcum*	Highly CO_2_-tolerant green alga with a growth rate optimum at 22°C that has been investigated for photoautotrophic lipid production	• *In vitro* oxygenation of multilayered cell-sheet-based bioartificial cardiac tissue	Constant illumination (500–700 lux, or 1313 ± 45 lux)	Haraguchi et al., 2017
				Illumination with an LED light source (30–170 μmol m^–2^ s^–1^)	Ota et al., 2015a,b
*C. pyrenoidosa*	Unicellular eukaryotic green algae of the genus *Chlorella*	Grows optimally at 37°C and pH 7.4.	• Implementation in the development of a photosynthetic artificial lung	Illumination with 400 W Hg-metal halide hybrid lamp (luminous flux of 28,000 lumens, color rendering index of 91, and chromaticity of 5200 K) and 300 W of plant grow lights	Basu-Dutt et al., 1997
*C. vulgaris*	Unicellular eukaryotic green algae of the genus *Chlorella*	Has emerged as a promising alternative feedstock and nutritional supplement with anticancer and anti-inflammatory effects. Optimal growth at 30°C, pH 8.2–8.7. Contains a large concentration of chlorophyll	• Photosynthetic oxygenation of explanted pancreatic tissue	Illumination for 30 min., LED lights (70,000 lux)	Yamaoka et al., 2012
			• Potential use as an oxygen-generating system to enhance radio- and photodynamic cancer therapy	Constant illumination, cool white fluorescent light (3,000 lux); Illumination for 2 h, red light diode (660 nm, 6,000 lux)	Morimoto et al., 1995; Qiao et al., 2020
*C. sorokiniana*	Unicellular eukaryotic green algae of the genus *Chlorella*	Potential for application in wastewater treatment and biodiesel production. Tolerates up to 42°C and shows high growth rate at 37°C	• Photosynthetic oxygen supply to encapsulated pancreatic islets	Constant illumination, fiber optic light source	Bloch et al., 2006; Eladel et al., 2019
				Constant illumination, tubular fluorescent lamp (100 μmol photons m^–2^ s^–1)^	Morimoto et al., 1995; Qiao et al., 2020
*S. elongatus*	Unicellular cyanobacterium of the genus *Synechococcus*	Has been thoroughly investigated as a candidate for photoautotrophic biosynthesis in diverse biotechnological applications, cultivation optimum at > 30°C	• Proof-of-principle of vertebrate-cyanobacterium symbiosis	Constant illumination with strong light	Agapakis et al., 2011
			• Intramyocardial photosynthetic oxygen supply to alleviate ischemia upon myocardial infarction	Constant illumination, plant fluorescent light bulbs	Cohen et al., 2017
			• Microalgae-gel patch for healing of chronic diabetes-associated wounds	Illumination in 6 cycle-period of 30 min., near-infrared light-emitting diode (620–660 nm, 0.5 W/cm^2^ power density)	Chen et al., 2020
			• Photoautotrophic biosynthesis	Constant illumination, white light (500 μmol photons ⋅ m^–2^ s^–1^); constant illumination, artificial cool white light (55 ± 0.5 μmol photons ⋅ m^–2^ ⋅ s^–1^)	Yu et al., 2015; Sarnaik et al., 2017

## Photosymbiosis in Nature

Photosynthesis mediates the conversion of solar energy into biomass, and has had an immense influence on geochemical and biological evolution since its advent ca. 2.4 billion years ago (Hohmann-Marriott and Blankenship, 2011). Photosynthetic light reactions take place within specialized membrane systems named thylakoids in cyanobacteria, algae and plants. These membranes harbor the main constituents of the photosynthetic electron transport (PET) chain, i.e., photosystem II, the cytb_6_f complex and photosystem I, which generate a protonmotive force that is used for the production of ATP. Strikingly, the initial steps of PET in photosystem II produce molecular oxygen as a by-product, which extracts electrons from water. These electrons are then used to fuel the PET (Nickelsen and Rengstl, 2013). Thus, PSII functions as a light-driven oxidoreductase, which comprises more than 20 subunits and numerous low-molecular-weight cofactors, including organic pigments like chlorophylls and carotenoids as well as essential inorganic constituents such as iron and manganese. Indeed, the last-named metal is essential for oxygen production, as it forms part of the Mn_4_O cluster that catalyzes photon-driven water oxidation (Shen, 2015).

Photosynthetic organisms, also known as phototrophs, use the chemical energy generated by the PET for the synthesis of carbohydrates, lipids and proteins, thereby producing the metabolites required to sustain not only their own survival but also that of the heterotrophs that feed on them. Among the most remarkable of ecological relationships is photosymbiosis—the stable mutualistic, extra- or intracellular, symbiotic association between a heterotrophic organism and a photosynthetic protist. Examples of photosymbiosis are found among sponges, cnidarians, flatworms, molluscs, ascidians and even some vertebrates. These organisms have evolved to establish and maintain symbiotic relationships by hosting photosynthetic bacteria, cyanobacteria or unicellular algae under a common principle of exchange, in which the symbiont provides its host with organic carbon metabolites produced through photosynthesis, while the host organism offers protection from predators and environmental hazards, plus a supply of inorganic compounds, such as carbon dioxide, which serve as metabolites for the photosynthetic organisms. Since such interactions result in the host’s acquisition of many of the benefits of photosynthesis (Melo Clavijo et al., 2018), these relationships have been described as a “domestication of photosynthesis that can result in atrophic independence (…) under the infinite source of solar energy” (Bailly et al., 2014), and it may cover up to 90% of the host’s energy requirements (Kühlmann, 1993), and an estimate of 50% of all marine photosynthesis (Bailly et al., 2014). Intriguingly, while the genetic mechanisms that have allowed the establishment of photosymbiosis have been actively studied (Melo Clavijo et al., 2018), the relevance of oxygen production within these relationships has only begun to be understood.

For instance, reef-building corals form an endosymbiotic relationship with dinoflagellate algae belonging to the genus *Symbiodinium*, which take up residence and remain photosynthetically active within the endodermal tissue of the cnidarian. The impact of this photosymbiotic association between algae and host is so great that it is considered to be the key to understanding the environmental problem of coral bleaching. This phenomenon, in which an initial photosynthetic impairment in the endosymbiotic algae, owing to loss of the photosynthetic pigments, leads to the subsequent expulsion of *Symbodinium* from the host, has been correlated with a response to thermal stress (Hill et al., 2014). Moreover, the presence and photosynthetic potential of *Symbodinium* spp. increases the ability of *Cassiopea* sp. polyps to cope with the effects of ocean acidification, as it may mitigate the combined effects of hypoxia and the drop in the pH of seawater (Klein et al., 2017). Interestingly, a recent study of the molecular mechanisms involved in adaptation to variations in oxygen concentrations in cnidarians identified conserved oxygen-dependent signal transduction mechanisms, similar to the transcriptional response mediated by hypoxia-inducible factor-1 (HIF-1) in mammals. Since corals are not susceptible to the rapid transition between hyperoxia during the day and intra-tissue hypoxia at night, the authors of this study proposed these invertebrate animals as an insightful model in which to investigate the evolution of regulatory mechanisms of oxygen homeostasis (Zoccola et al., 2017).

Marine metazoans are also well-known to have taken advantage of photosynthesis, and some such symbiotic relationships have become essential for survival. For example, the green hydra *Hydra viridissima* evolved into an endosymbiotic organism by incorporating *Chlorella* algae, which allow it to survive periods of starvation and also supply these animals with an advantageous mode of camouflage in their aquatic environment (Ishikawa et al., 2016). Even though the physiological role of photosynthetic oxygen in this relationship is still unclear, it has been shown that the presence of the algae affects asexual reproduction and plays a major part in the sexual differentiation of the host (Habetha et al., 2003). Another example of a photosymbiotic relationship is the one between the tidal acoel flatworm *Symsagittifera roscoffensis* and the green alga *Tetraselmis convolutae*. Here, it has been estimated that the number of algal cells within each adult flatworm may reach 40,000 (Doonan and Gooday, 1982), and together they maintain a tight equilibrium between photosynthesis and respiration, which allows each species to profit from the other’s metabolites. In fact, both the animal’s survival and its capacity to develop to maturity are directly dependent on the presence of the algae (Bailly et al., 2014).

Photosymbiosis may even provide a more favorable microenvironment or more specialized adaptations for some higher organisms. For instance, the photosynthetic capacity of insect-induced bud galls in acacia plants contributes to their maintenance and growth, partly by providing oxygen to the insect larvae, but also by compensating for the carbon-sink effect induced by the galls on the host plant (Haiden et al., 2012). Even more surprising is the example of the salamander *Ambystoma maculatum*, which is the first vertebrate found to harbor intracellular photosynthetic endosymbionts in its tissues throughout its entire lifespan, and provides an especially informative example of the benefits of co-existence with a photosynthetic organism. In the late nineteenth century, Henry Orr first described the presence of single-celled *Oophila amblystomatis* algae in the immediate vicinity of embryos of the salamander. But it was only a few years ago that Ryan Kerney discovered that the algae were in fact inside the embryos, and not just within the jelly sacs surrounding them (Kerney et al., 2011). Moreover, Kerney’s electron microscopic analysis showed that, at points of contact, mitochondria in the salamander cells form clusters adjacent to the algal symbiont, further supporting the hypothesis that photosynthetic oxygen could be directly coupled to mitochondria energy production, which was later proven experimentally (Graham et al., 2013). Studies of the importance of the photosymbiotic relationship between these organisms have further shown that the incorporation of the algae into the salamander embryos elevates the oxygen content of the water in their immediate vicinity (Graham et al., 2013; Small et al., 2014), which in turn promotes the hatching of the embryos and protects them against infections (Tattersall and Spiegelaar, 2008; Kerney, 2011). Besides, it appears that the algae help to clear the nitrogen-rich waste released by the embryo and, even more fascinatingly, they become dormant when removed from their host (Gilbert, 1942, 1944). While the precise mechanism and time point of incorporation of the algae remain to be elucidated, it has been suggested that the symbiotic algae could also be passed directly to the offspring’s jelly sacs, since algae have been found in the oviducts of adult female salamanders, which is where the embryo-encompassing jelly sacs form (Kerney et al., 2011; Burns et al., 2017).

The fact that *O. amblystomatis* can stably survive inside the salamander suggests that these unicellular microalgae manage to evade destruction by the vertebrate host’s immune system. Attempts to reproduce this photosymbiont-vertebrate interaction under controlled experimental conditions have been undertaken by two independent research groups, who injected photosynthetic *Synechococcus elongatus* PCC 7942 cyanobacteria (Agapakis et al., 2011) and *Chlamydomonas reinhardtii* (*C. reinhardtii*) microalgae (Alvarez et al., 2015), respectively, into zebrafish embryos. In the first study, the authors showed higher biocompatibility of the cyanobacteria (relative to *E. coli* cells) with the zebrafish embryo. They also showed that the photosynthetic organisms were capable of surviving and proliferating inside mammalian cells when cultured under illumination. In the second study, Alvarez et al. (2015) established viable plant-vertebrate chimeras, and demonstrated that both the injected algae and the zebrafish embryo/larvae could survive for several days after injection, during which time no significant innate immune response against the “photosymbiont” nor any developmental impairment of the host was observed. The results presented in both studies suggest that, up to a certain level, photosynthetic organisms are well tolerated as foreign bodies by the developing zebrafish. Lastly, two studies have attempted to generate plant-animal hybrid cells following strategies based on induced phagocytosis (Jones et al., 1976) and cell fusion (Wada et al., 2016), respectively. However, no stable photosynthetic animal cell line has been created so far.

## Photosynthetic Approaches for Tissue Oxygenation

**FIGURE 1:**
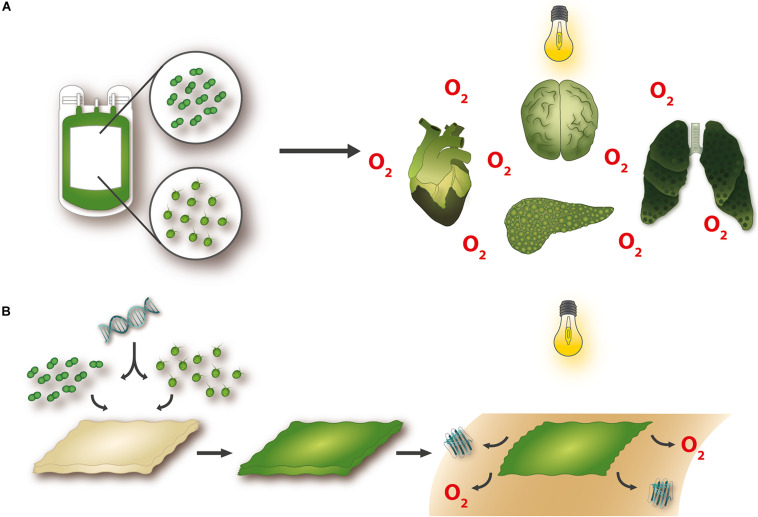
Potential applications for photosynthetic technologies. Novel approaches to organ oxygenation have shown that microorganisms with the capacity to produce oxygen when stimulated with light can improve organ functionality and survival in the absence of blood perfusion **(A)**. Furthermore, the availability of genetic tools makes it possible to construct transgenic photosynthetic organisms that bioactivate tissue-engineered materials and confer upon them the potential to simultaneously and steadily release oxygen and functional recombinant molecules, such as growth factors **(B)**.

The direct dependence of animal life on photosynthesis was irrefutably demonstrated in 1772 when Joseph Priestley showed that a mouse would die when it was placed in a sealed compartment but survived if a plant was introduced as well (Priestley, 1772). Almost 200 years later, in a pioneering study, Boerema et al. (1960) demonstrated the feasibility of effective oxygen delivery to tissues in the absence of blood cells, thereby showing that the cardiovascular system could be replaced by an alternative oxygen delivery system. Taking this into account, together with the examples of photosymbiosis in nature, it is intriguing (to say the least!) to investigate whether the phenomenon can be employed to confront the clinical challenges associated with tissue hypoxia (Figure 1).

The first experiments that explored the idea of using microalgae for this purpose go back to 1994, when preliminary studies were conducted toward the development of a photosynthesis-based life-support system for individuals at risk of acute respiratory failure (Basu et al., 1994). Respiratory failure is a condition in which the respiratory system cannot provide for adequate gas exchange, and consequently results in hypoxemia and/or build-up of carbon dioxide. In patients with severe pulmonary dysfunction and end-stage lung diseases, extracorporeal membrane oxygenation (ECMO) has since evolved as a rescue therapy to maintain oxygenation and CO_2_ removal. While most patients requiring ECMO are critical-care patients in intensive care units, some patients requiring long-term ECMO therapy are effectively immobilized owing to the bulkiness of the device, which impairs their quality of life (Makdisi and Wang, 2015). In two subsequent studies (Basu et al., 1994; Basu-Dutt et al., 1997), Basu and collaborators set out to develop a light, portable and energetically efficient “photosynthetic artificial lung” by combining an ECMO unit equipped with an external collector of light energy with a photobioreactor for the cultivation of the green alga *Chlorella pyrenoidosa* (*C. pyrenoidosa*). They showed that the algae were capable of photochemically producing O_2_ and removing CO_2_ and went on to optimize parameters affecting their photosynthetic efficiency, such as culture medium, cell density, substrate availability and illumination. Then they developed a prototype with which to test the life-support system *in vitro* by interfacing it with blood via a gas-transfer membrane. While the results obtained were regarded as promising, since the algae were able to supply O_2_ and remove CO_2_ at more than half the rate required for physiological applications, the authors concluded that major advances in the design and customization of the photobioreactor would be required before the technology was mature enough for clinical use.

Oxygen deprivation is also a major concern in the field of organ transplantation, in particular for pancreatic tissue. Type 1 diabetes is an autoimmune disorder in which specific loss of pancreatic β-cells and consequent insulin deficiency result in hyperglycemia (Atkinson et al., 2014). In this context, pancreas transplantation is currently the treatment option of last resort for the restoration of glucose homeostasis and avoidance of life-threatening diabetic hypoglycemia or ketoacidosis. However, its success depends crucially on the maintenance of organ viability during the interval between donation and transplantation (Giorgakis et al., 2018). To sustain oxygen saturation in the absence of blood perfusion, and maintain functionality over this period, Yamaoka et al. adopted an experimental approach using the photosynthetic microalga *Chlorella vulgaris* (*C. vulgaris*) (Yamaoka et al., 2012). In their study, pancreases removed from donor male LEW rats by laparotomy were stored in a gas-permeable, porous pouch containing live algae until implantation. According to the results reported, tissue viability in heterotopically transplanted rat pancreases could be maintained for 3 h after loss of blood perfusion. Moreover, the group of diabetic rats that received organs that had been preserved in this way showed normal glucose regulation and extended survival after surgery, relative to animals treated with pancreatic transplants that had been maintained statically in cold storage, which is currently the standard form of preservation in clinical practice. Most remarkably, in the same study, the authors demonstrated the ability of microalgae to improve the oxygenation of the internal organs of adult male Sprague-Dawley rats with induced respiratory insufficiency.

Thereafter, the idea of oxygen generation using the photosynthetic capacity of microalgae was taken up by Bloch et al. (2006) as a way to overcome severe hypoxia and cell dysfunction caused by the immunoisolation of pancreatic islets and the interruption of vascular connections upon implantation. Pancreatic islet transplantation is an experimental treatment that has been shown to restore β-cell function, provide glycemic control and achieve sustained insulin independence in diabetics. As yet, its success is directly dependent on the survival of significant numbers of the engrafted pancreatic islets (Gibly et al., 2011). Bloch et al. (2006) sought an experimental means of avoiding this problem by co-encapsulating pancreatic islets isolated from a murine pancreas with *Chlorella sorokiniana* (*C. sorokiniana*) in alginate. *C. sorokiniana* was first shown to produce a significant amount of oxygen in the light, and to survive in the vicinity of mammalian cells. Subsequently, under oxygen-free conditions, co-encapsulated islets were shown to respond to glucose stimulation upon illumination, unlike encapsulated islets alone or co-encapsulates kept in the dark. Furthermore, photosynthetic oxygen supply evoked a higher level of insulin expression than normoxic perfusion, which strongly suggests that photosynthetic oxygen generation by microalgae could potentially bypass hypoxia in bioartificial pancreatic tissues.

Photosynthesis has also been explored as an alternative source of oxygen for the restoration of cardiac tissue subjected to acute ischemia caused by coronary artery disease and myocardial infarct. Cohen et al. reported an unprecedented method for correcting myocardial ischemia, which involves intramyocardial oxygen delivery by *Synechococcus elongatus* (*S. elongatus*), a unicellular cyanobacterium with a very high photosynthetic capacity (Cohen et al., 2017). After demonstrating the feasibility of cardiomyocyte-*S. elongatus* co-culture under (mammalian) physiological conditions, they tested the approach under hypoxic conditions and found that cellular metabolism was indeed enhanced when oxygen production was stimulated by light. Then, the group measured the effect of *S. elongatus* on tissue oxygenation in an acute myocardial infarction model using male immunocompetent Wistar rats. The cyanobacteria were delivered by intramyocardial injection, and ischemia was induced through occlusion of the left anterior descending (LAD) coronary artery. By inducing photosynthetic activity through the exposure of *S. elongatus* to light, they were able to rescue the myocardium from acute ischemia, and showed that this effect was associated with an increase in tissue oxygenation, and improved cellular metabolism, ventricular function and performance, relative to rats treated by injection of non-illuminated *S. elongatus* and saline controls (Cohen et al., 2017). Importantly, *S. elongatus* was found to be non-toxic and non-pathogenic in recipient rats, while in a follow-up study, *S. elongatus* failed to evoke an immune response when injected intravenously into Wistar rats, making this an intriguing and potentially feasible approach for the treatment of myocardial ischemia (Williams et al., 2020).

Finally, a very recent study demonstrated the use of photosynthetic microalgae to potentiate radiotherapy in tumor treatment (Qiao et al., 2020). In this case, *C. vulgaris* microalgae were coated with membrane components purified from erythrocytes to prevent clearance by macrophages, and then delivered into orthotopic breast cancer tumors in female Balb/c mice either directly or via intravenous injection. Increased levels of blood oxygen were observed in the tumor region when photosynthesis was induced by illumination with red light, and in association with X-ray irradiation this measure effectively prevented tumor growth. Moreover, the algae enhanced the therapeutic effect of combined radiation and photodynamic therapy by releasing chlorophyll-derived ROS and reducing the levels of the angiogenic factors HIF1α and VEGF, thereby promoting cancer-cell apoptosis and inhibiting tumor angiogenesis and proliferation.

## Photosynthetic Biomaterials for Tissue Engineering and Regeneration

**FIGURE 2:**
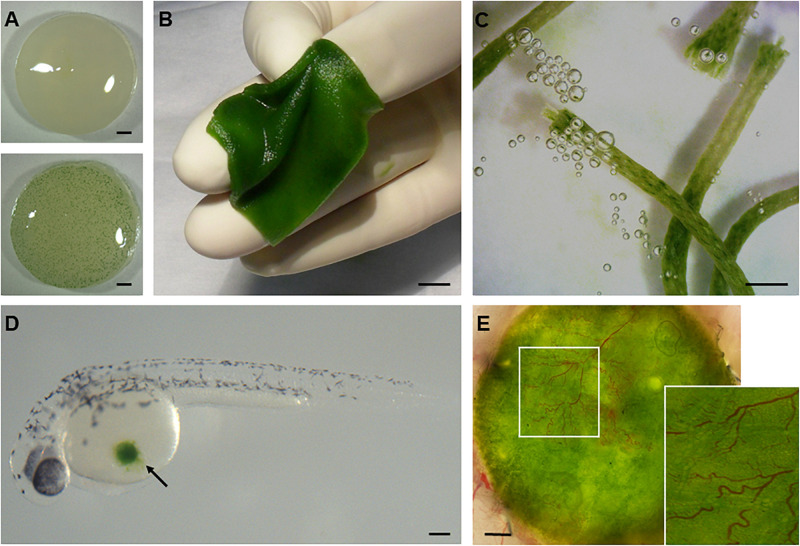
Development of photosynthetic biomaterials. The combination of photosynthetic microorganisms such as cyanobacteria **(A)** and green algae **(B–E)** with biomedical devices routinely used in clinical practice, such as dermal scaffolds **(B)** or suture threads **(C)**, has enabled the development of biomaterials that are capable of producing oxygen *in situ*. Moreover, the biocompatibility of these organisms with animal life has been demonstrated both *in vitro* under co-cultivation conditions with mammalian cells and *in vivo* in several vertebrate models like zebrafish **(D)** and mice **(E)**. Scale bars represent 1 mm in **(A,C,E)**, 1 cm in **(B)**, and 0.2 mm in **(D)**.

Photosynthetic microorganisms have found a particular niche within the field of tissue engineering and regeneration (Figure 2), where the regenerative potential of biomaterials is primarily limited by hypoxia (Iyer et al., 2007; Malda et al., 2007; Colom et al., 2014). In this context, Hopfner et al. (2014) first described the development of photosynthetic biomaterials for *in-vitro* tissue engineering in 2014, using *C. reinhardtii* as a platform for photosynthesis, together with collagen-based dermal scaffolds. In their study, two- and three-dimensional cell co-cultures consisting of *C. reinhardtii* and murine fibroblasts were established to verify the biocompatibility of the heterologous cells. They went on to demonstrate that illumination resulted in a decreased hypoxic response in fibroblasts, owing to the presence of photosynthetic oxygen. This first generation of photosynthetic biomaterials caused a paradigm shift in tissue engineering, by decreasing tissue hypoxia independently of vascularization, perfusion or external oxygen supply. In the following year, Schenck et al. (2015) verified the feasibility of transferring this *in vitro* concept into living organisms by implanting the photosynthetic biomaterials in murine full-skin defects. For this, they developed an encapsulation technique that enabled them to seed and maintain the algae inside the dermal scaffolds, which promoted both proliferation of the algae and the release of photosynthetic oxygen, as confirmed *in vitro*. *In vivo*, they reported high levels of vascularization when these alga-containing implants were exposed to light, and implantation of the photosynthetic scaffolds into female *nu/nu* athymic mice failed to trigger an immune response. Taking the approach one step further, and motivated by the idea that genetic modification might enhance the regenerative potential of *C. reinhardtii*, Chávez et al. introduced the concept of photosynthetic gene therapy by incorporating transgenic microalgae that secrete recombinant human growth factors, in addition to oxygen, into the scaffolds (Chávez et al., 2016). In this study, they also used immunocompetent hairless female Skh1 mice to prove the safety and biocompatibility of the algal cells over an extended length of time. Finally, the concepts of photosynthetic biomaterials and photosynthetic gene therapy were implemented by the same group to create photosynthetic sutures for the local delivery of oxygen and recombinant growth factors to incisional wounds (Centeno-Cerdas et al., 2018).

Algae other than *C. reinhardtii* have also been investigated for photosynthetic oxygen release in tissue engineering applications. *Chlorococcum littorale* (*C. littorale*) has been used in the generation of three-dimensional (3D) bioartificial cardiac tissues. 3D cell culture systems, such as cell-sheet-based approaches, have become a major focus in the field of cardiac tissue engineering and regeneration, which aims to grow functionally organized tissue replacements by providing a microenvironment which replicates that of native cardiovascular structures (Cohen et al., 2017). However, the hypoxic microenvironment created when multiple cell sheets are stacked—in the absence of a vascular network—limits the thickness of viable layered 3D tissue to 40–80 μm (Shimizu et al., 2006), which presents a major obstacle to their development and deployment. To overcome this drawback, in 2017 Haraguchi et al. (2017) explored the idea of using *C. littorale*, a unicellular green spheroidal alga with a high capacity for CO_2_ fixation and a significant photosynthetic potential (Satoh et al., 2002). By co-culturing multilayer cell sheets composed of rat cardiomyocytes together with *C. littorale*, they were able to bioengineer significantly thicker (160 μm) viable tissue consisting of up five cell sheets. Even more remarkably, the authors found significantly lower glucose consumption and lactate production in multilayered sheets co-cultivated with the algae. They attributed this to more effective ATP production, in association with aerobic respiration driven by the increased oxygen supply provided by the algae. Furthermore, significant decreases in both cell-sheet delamination and the release of creatine kinase—both common indicators of muscle cell damage—were detected upon histological analysis of the co-cultured cell sheets. Taken together, these findings indicate the potential of photosynthetic oxygen supply in the context of the assembly of bioartificial cardiac sheets that could reverse cardiac damage.

Remarkable advances have also been made in the encapsulation of microalgae for bioactivation of tissue engineering constructs. For instance, Lode et al. (2015) demonstrated the feasibility of encapsulating microalgae in three-dimensional alginate scaffolds with a geometry fabricated by computer-based plotting of the optimal fiber network through a rapid prototype method. They used *C. reinhardtii* as a model organism to show that, upon immobilization in the alginate−based matrix, the algal cells survived the plotting process and could be cultivated within the hydrogel matrix. Moreover, they could be co-incorporated into the network together with human osteosarcoma cells during the fabrication procedure. Later, Krujatz et al. (2015) extended this strategy by investigating the growth and viability of microalgae within 3D-plotted alginate hydrogels and suspension cultures at different temperatures and under various illumination conditions. Interestingly, these authors demonstrated that cell viability was significantly higher when the algae were exposed to a 14/10 h light/dark cycle rather than continuous illumination. Moreover, microalgae embedded within the hydrogel in a highly structured pattern grew at more stable rates than in suspension cultures—irrespective of the illumination conditions, and even at non-ideal temperatures for the algae (26–37°C). Lastly, in an attempt to enhance the biotechnological production of secondary metabolite production, the same research group went one step further and developed a way to integrate cell cultures of a higher plant (basil) into 3D hydrogel scaffolds (Seidel et al., 2017), thereby achieving a new milestone in what they named “green bioprinting.”

During the preparation of this manuscript, a new study appeared which introduced a novel biomedical application of photosynthetic microorganisms as an oxygen source to promote the healing of skin grafts, and chronic diabetes-associated wounds (Chen et al., 2020). The authors developed a patch dressing containing living *S. elongatus* PCC7942 inside hydrogel beads, with which the wound could be covered, thus allowing gas exchange without eliciting a proinflammatory macrophage response. Under red light irradiation, this “alga-gel patch” produced oxygen, which rapidly and deeply penetrated the skin of immunodeficient Balb/C mice, thereby promoting wound healing and angiogenesis in full-thickness and skin-flap injuries.

## Transgenic Photosynthetic Organisms

Microalgae and cyanobacteria have significant biotechnological potential, owing to their ease of cultivation, fast growth rates, amenability to genetic manipulation, and the availability of complete genome sequences for many species (Rasala and Mayfield, 2015; Kumar et al., 2019). Furthermore, most of these photosynthetic microorganisms have been accorded “GRAS” (generally regarded as safe) status, since they do not carry human pathogens such as viruses, or prions or bacterial endotoxins (Fields et al., 2020). Thus, their potential can be exploited without the risk of pathogen transmission, and they can even be used for direct applications, such as dermal dressings (Schenck et al., 2015; Chávez et al., 2016), oral vaccines, food additives or nutritional supplements (Gregory and Mayfield, 2014; Gateau et al., 2017; Mysliwa-Kurdziel and Solymosi, 2017). Photosynthetic platforms offer versatility of product targeting, since they merely require the engineering of a transgenic strain to enable the production of a specific product (Mayfield and Golden, 2015). Due to their autotrophic nature, they are viewed as a cost-effective and environmentally friendly platform that offers a number of advantages over alternative approaches to the production of recombinant proteins such as cytokines (Rosales-Mendoza, 2016), growth factors (Wijffels et al., 2013; Rasala and Mayfield, 2015) and other promising bioactive molecules such as biomaterials (Molino et al., 2016) and enzybiotic proteins, i.e., enzymes designed to have an antibiotic function (Stoffels et al., 2017). The methodology and molecular tools required to generate transgenic photosynthetic organisms capable of producing recombinant biomolecules are now so well established that, to date, more than 45 recombinant proteins have been produced in green algae such as *C. reinhardtii*, and many more in cyanobacteria (Vijayakumar and Menakha, 2015; Singh et al., 2017). In particular, production of biomolecules in microalgae ensures appropriate translational processing, thereby retaining protein functionality (Scaife et al., 2015), while the option of extracellular secretion targeting makes the purification process less expensive and more straightforward (Ramos-Martinez et al., 2017; Baier et al., 2018). Above all, strategies that allow for the induction of transgene expression in response to heat (Schroda et al., 2002), light (Baek et al., 2016), ethanol (Lee et al., 2018), or hypoxic conditions (Iacopino et al., 2019), which are a current focus of research, could be of great benefit for biomedical applications.

While research on green molecular farming has mainly focused on *Chlamydomonas*, other microalgae, such as the diatom *Phaeodactylum tricornutum*, have shown greater potential with respect to post-translational modifications and the assembly of multiple recombinant protein subunits. Examples such as the synthesis of functional human IgG antibodies against hepatitis B virus (HBV) surface proteins (Hempel et al., 2011; Hempel and Maier, 2012) and Marburg virus nucleoprotein (Hempel et al., 2017) have shown that this inducible expression system can efficiently produce and secrete functional monoclonal antibodies at levels of up to 9% of total soluble protein, with the further advantage of safer and easier downstream processing compared to mammalian cell lines. On the other hand, the advantages of cyanobacteria systems as biotechnological platforms for recombinant protein production are beginning to attract researchers to the field. In particular, the euryhaline unicellular cyanobacterium *Synechococcus* sp. PCC 7002 (*Syn 7002*) has recently garnered attention as an industrial host and a novel biomedical tool owing to its fast growth rate, and its tolerance to high light levels, high salt and a wide range of temperatures make it an excellent platform for biotechnological applications. Moreover, its genome has been completely sequenced, while ongoing research on this organism is rapidly expanding the molecular toolkit available for genetic manipulation. This now includes promoter design, reporter proteins, selection strategies and overproduction protocols for recombinant protein expression (Ludwig and Bryant, 2012; Ruffing et al., 2016). Promising applications for this cyanobacterial strain include its use to produce biogenic polyphosphate nanoparticles to treat and prevent inflammatory gastrointestinal diseases (Feng et al., 2018a,b, 2019), and hyaluronic acid, a natural polymer with a broad range of cosmetic and biomedical applications (Zhang et al., 2019). Other attractive *Synechococcus* strains, such as *Synechococcus elongatus* PCC 7942 and the more recently characterized *Synechococcus elongatus* UTEX 2973 (Yu et al., 2015), are already being used for the synthesis of bioactive molecules such as heparosan, a pharmaceutical precursor of heparin, and glycosaminoglycans (Sarnaik et al., 2019), including heparan sulfate, chondroitin sulfate and hyaluronic acid, which play key roles in tissue maintenance, repair and regeneration (Melrose, 2016).

Still more impressive is the number and variety of applications that have been found for alga- and cyanobacteria-derived secondary metabolites. The list now includes more than 140 different molecules including polysaccharides, amino acids, carotenoids, isoprenoids, sterols, polyphenolic compounds, fatty acids and halogenated compounds, which are pharmaceutically active when employed as antiviral, antimicrobial and anti-inflammatory agents (Riccio and Lauritano, 2020). Besides their value as nutraceuticals, some have shown promising results as anticarcinogenic and antitumor compounds for several multidrug-resistant and advanced cancer types. A recent review has also pointed out the potential of microalgae and cyanobacteria in thalassotherapy (Mourelle et al., 2017), in particular because of the enriched spectrum of biologically active substances they can generate, which are relevant in the context of skincare, rejuvenation and regeneration. However, despite their recognized innate advantage as autotrophic organisms, the full extent of their potential for photosynthetic oxygen production in biomedical settings, and the valuable properties of the compounds they have been shown to synthesize, remains to be explored. For a more detailed consideration of the potential biomedical and pharmaceutical applications of bioengineered cyanobacteria and algae, the authors recommend the following review articles (Mayfield and Golden, 2015; Singh et al., 2017; Dehghani et al., 2019).

As mentioned above, genetically modified photosynthetic microorganisms could boost the regenerative potential of photosymbiotic approaches by combining photosynthetic oxygen supply with the local release of molecules that promote tissue regeneration. Three studies, in particular, have shown how photosynthetic microorganisms could be used to stimulate wound healing by creating an actively supportive microenvironment at the wound site itself. In the first, Chávez et al. (2016) engineered a genetically modified *C. reinhardtii* strain to constitutively express the human vascular endothelial growth factor VEGF-165 (hVEGF) with the aim of boosting the pro-angiogenic effect of photosynthetic dermal scaffolds. The authors showed that overexpression of recombinant hVEGF by these algal cells following their implantation in immunocompetent mice promoted endothelial cell migration and blood vessel formation, whereas blood vessel ingrowth was observed after injection of the transgenic microalgae into the yolk sac of zebrafish embryos. In the second study, Centeno-Cerdas et al. (2018) tested the hypothesis that seeding of absorbable sutures with genetically modified *C. reinhardtii* would promote wound healing immediately after surgical closure. Here, the authors demonstrated that the photosynthetic sutures were indeed capable of secreting significant amounts of bio-functional recombinant hVEGF, platelet-derived growth factor B (hPDGF-B) and stromal cell-derived factor-1 (hSDF-1), as well as releasing substantial levels of oxygen upon exposure to light (Centeno-Cerdas et al., 2018). Finally, Jarquín-Cordero et al. (2020) recently established an optimized platform for the secretion of alga-derived human growth factors and described the potentiating angiogenic effect of the combinatorial application of *C. reinhardtii* transgenic strains. All these results demonstrate the significant potential of transgenic photosynthetic organisms for the production of defined therapeutics for biomedical applications in the future.

## Outlook

Oxygen is not only vital for cellular respiration in untold numbers of living organisms including ourselves, it also plays a key role in the healing and regeneration of their tissues. Conversely, effective and safe oxygen perfusion represents one of the greatest challenges in the fields of organ transplantation, tissue engineering and chronic wound management. The many examples of photosymbiosis found in nature are the most compelling argument for the notion that the induction of photosynthesis in human tissues offers a feasible approach to the avoidance of tissue hypoxia in clinical settings. This has led to the exploration of photosynthesis as an alternative means of meeting the metabolic requirements of cells and tissues in the context of a variety of medical conditions, including respiratory failure, restoration of pancreatic function, cardiac ischemia and tissue regeneration.

Moreover, the potential of photosynthetic biomaterials for tissue engineering has just begun to be revealed. While all the examples mentioned in this review demonstrate that bioactivation of scaffolds with photosynthetic microorganisms can in principle provide a constant source of oxygen supply, some interesting findings suggest that they could further improve other parameters within established cell-culture systems, such as the removal of toxic metabolites or the secretion of beneficial secondary metabolites. Also, the parallel development of novel photosynthetic-cell encapsulation and immobilization technologies could further enhance photosynthetic cell therapy, as it should lead to general improvements in cultivation methods. Novel methods to combat tissue hypoxia in the field of tissue engineering are desperately needed, and if oxygen-producing photosynthetic biomaterials were able to fulfill this task, they might provide a means of accelerating the clinical application of bioartificial tissues. Besides, as already mentioned, the possibility of combining photosynthetic approaches with genetic engineering tools for transgene expression could enhance their clinical impact by providing for the local release of other therapeutic molecules, in addition to oxygen. Although proof-of-concept protocols have been described, well-established, standardized and efficient molecular tools that support a constructive biotechnological platform still have to be developed. Detailed characterization and a deeper understanding of specific promoters will be required to ensure that foreign gene expression can be precisely modulated in response to clinical needs.

Although the translation of these photosynthetic biomedical concepts to the clinic remains to be achieved, ongoing research shows how photosynthetic approaches may diversify and rapidly progress to breakthroughs in their clinical use in several fields of medicine. For instance, the induction of local photosynthesis could have a great impact on the economics of healthcare and supply currently unmet clinical needs for the stable oxygenation of partially or non-perfused organs. Indeed, some of the results discussed above suggest that photosynthetic oxygen supply could, in fact, enhance organ performance to levels above physiological normoxia, hereby opening possible medical applications for photosynthetic biotechnologies beyond the mitigation/rescue of ischemic conditions. However, to mimic photosymbiotic relationships for medical purposes, a better understanding of the cellular and molecular mechanisms that govern this process is urgently required. In this respect, one promising route to the successful implementation of this concept would be to capitalize on the broad biodiversity observed among photosynthetic organisms and carry out a massive screen for candidates that display the combinations of traits demanded by each different clinical application. Among the key parameters to be considered are features such as the appropriate shape, size and life cycle, as well as the light spectrum/intensity requirements for photosynthesis, and optimal growth temperature. Then, by providing a repertoire of safety-tested photosynthetic organisms with diverse optimal growth requirements and attributes, a better match could be found for every ischemic pathology, given that—besides the lack of oxygen—issues such as excess of carbon dioxide, nutrient deprivation and accumulation of cellular waste may be relevant. For instance, exploring the applicability of other autotrophic organisms that have already evolved to maintain a photosymbiotic relationship with an animal host, such as dinoflagellates, could be a smart strategy in the search for valuable candidates for biomedical approaches. It is important to point out that, because oxygen production in photosymbiotic approaches relies on constant illumination, the development of novel illumination technologies to address issues such as heat relief and light penetration into different tissues presents a further set of challenges. Also, because most human tissues are not adapted to cope with light radiation, the possible role of phototoxicity in this context remains to be clarified. Nevertheless, the examples listed in Table 1 demonstrate that a customized illumination strategy for induced photosynthetic oxygen production may be developed for both internal and external human organs.

Whether one considers cell or tissue-level therapies, or applications to organ transplantation, the clinical success of photosymbiosis will strongly depend on the ability of the host immune system to handle the foreign photosynthetic symbionts. Along with the systemic use of immunomodulatory drugs to induce tolerance, other standard technologies could be explored in more detail and adapted appropriately, including the use of inert biomaterials to encapsulate implanted heterologous cells and circumvent the host’s immune response (Ashimova et al., 2019). In this context, alginate has demonstrated to be effective in protecting different cell types (Carroll et al., 2019; Ghanta et al., 2020) upon implantation, as well as more complex structures like pancreatic islets (Barkai et al., 2016). Besides, both microalgae and cyanobacteria could be genetically modified to locally release immunomodulatory factors, in addition to the compounds many of them already produce (Riccio and Lauritano, 2020), or as recently described, engineered to be coated with immunocompatible envelopes (Qiao et al., 2020). However, it would be far more interesting to search for immunotolerant photosynthetic microorganisms capable of establishing long-term relationships with humans. As discussed previously in this work, this idea is supported by evidence which indicates that vertebrate immune responses to photosynthetic organisms, even after systemic injection (Williams et al., 2020), are mild or non-existent. It is conceivable that the mammalian immune response might have evolved in the absence of the need to recognize photosynthetic cells as foreign entities (Wheeler et al., 2008). In addition, neither critical pathogen-associated molecular patterns compounds that are recognized by the native immune system (e.g., endotoxic LPS) nor toxic compounds are present in most of the photosynthetic microorganisms (Durai et al., 2015; Rosales-Mendoza et al., 2020). This raises hopes for the feasibility of establishing a photosymbiotic relationship resembling those utilized by reef-building corals, flatworms and adult salamanders. Even so, further *in vitro* and *in vivo* studies should address the detailed molecular mechanisms that govern this propitious immunotolerance between animals and photosynthetic cells. Other key questions that still need to be addressed concern the ability of photosynthetic biomaterials to fulfill all the local metabolic oxygen requirements, and the basic issue of the safety of this approach in human patients. In order to answer this last question, the first clinical trial (NCT03960164) has recently begun. However, no data have yet been released. Finally, a multidisciplinary approach based on close collaboration between basic scientists, clinicians and engineers will be required for the success of this approach; furthermore, owing to the possible implications of human photosynthesis beyond medicine, the transition from basic scientific research into clinical practice will require input from experts in public policy, ethics and economics.

## Author Contributions

MC: conceptualization, investigation, project administration, and writing—original draft. NM: investigation and writing—original draft. TS: resources and funding acquisition. JE: conceptualization, visualization, writing—review, and editing. JN: conceptualization, writing—original draft, resources, and funding acquisition. All authors contributed to the article and approved the submitted version.

## Conflict of Interest

JE was the founder and VP of Technology at SymbiOx Inc. This startup did not provide any financial support to this work but is closely related to some topics of this manuscript. In addition, he was currently supervising an ongoing clinical trial (NCT03960164) to prove the safety of the implantation of photosynthetic scaffolds in wounds that result from trauma injuries. The remaining authors declare that the research was conducted in the absence of any commercial or financial relationships that could be construed as a potential conflict of interest.
